# Swindlers

**Published:** 1869-04

**Authors:** 


### Swindlers.

### HOW SHARPERS TAKE ADVANTAGE OF THE UNWARY.

We have a large collection of circulars, pamphlets, newspapers, cards and the like, sent us by our subscribers, who ask us to give an opinion of them—to tell them whether the advertisers are reliable people or not, and whether their medicines and receipts are what their proprietors claim for them.

Some of these documents are curious to look upon, and exhibit an amount of cunning and intrigue startling to behold. Others will disgust one with their obscenity and astonish you to witness the depth of degradation to which the human species will descend, in order to secure to themselves profit without labor.

We have before us the card of an exceedingly benevolent gentleman, who styles himself "Dr. John B. Ogden," of 42 Cedar street, New York. This individual publishes to the world that he will forward, *free of charge*, a recipe to any young man who is suffering from "seminal weakness," his "only object in so doing being to benefit the unfortunate," &c. Of course, thousands of young men would at once respond to so generous an offer, and doing so, receive by return mail, a circular containing the desired recipe. Take this formula to the druggist, and he will tell you that the two first articles are unknown to him or the Dispensatory. What will you do? Precisely what the knowing doctor supposed you would do! Send to him "$3.03 for the medicine all ready prepared, if your druggist is unable to supply you." Easy enough you see. Then, this marvelously benevolent fellow offers his medicine precisely at cost, $3.03. The sum is so small, that you at once remit, and are—*swindled!*

Our readers have doubtless seen the advertisement of Thos. Chapin, M. D., of New York. This gay and festive chap is a dealer in "French cream for bald heads and bare faces," but has neglected to apply his remedy to his own case, and a more "bare faced" swindler does not exist anywhere. He will also send you a circular announcing that he has a "curative instrument" for spermatorrhœa, and "Dr. Bronson's infallible French monthly pills," at $5 per box, "which no married female should be without." He has a "hair-up-rooter," and another compound to make it grow again. Will remove your freckles, perfume your breath, give you "a moustache in one week," and supply you with

"love powders." He's a genius, is Dr. Chapin. Send him your money, by all means, and be—*swindled.*

"A lady who has suffered for years from Deafness, Catarrh and Scrofula, was cured by a simple remedy. Her gratitude prompts her to send receipts free of charge to any one similarly afflicted." Poor woman—how she must have suffered—wonder whether she ever had the measles? But by writing to Hoboken, N. J., she will supply you with the receipt and an announcement that she "keeps the medicine all ready prepared for $5." Send for it, why not? and be—*swindled.*

"Rev. Edward A. Wilson," of New York, made a very narrow escape from consumption —what a pity!—"was upon the very verge of the grave"—dear me! Will send a recipe, too, *free of charge*, and tell you how *he* was cured. *His* medicines come from South America, so that our druggists are unable to supply them. But the dear old man is unwilling that you shall die for the want of them, so he will supply you for $5. Send for them, certainly. After you have tried several bottles, and are no better, it will surely cure you of being—*swindled.*

---

HINTS TO MOTHERS AND NURSES.—There is reason to believe that not a few of the apparently unaccountable cases of scrofula among children proceed from the habit of sleeping with the head under the bed-clothes, and so inhaling air already breathed, which is further contaminated by exhalations from the skin. Patients are sometimes given to a similar habit, and it often happens that the bed-clothes are so disposed that the patient must necessarily breathe air more or less contaminated by exhalations from his skin. A good nurse will be careful to attend to this. It is an important part, so to speak, of ventilation.

---

NEXT TIME.—We have an excellent article from the pen of Dr. HAYS, on "colds," received too late for this number. We will give it to our readers in the July number.

We will also have an improvement in the "make up" of the BISTOURY, giving the different departments—Communications, Domestic Medicine, Correspondence, Medical Items and News, Editorial Chit Chat, Housekeeper, &c., better positions, and *will keep them there*, so that they can be readily referred to.

